# Parasites of *Astyanax lacustris* (Pisces, Characiformes) from Brazilian streams

**DOI:** 10.1590/S1984-29612024025

**Published:** 2024-06-17

**Authors:** Bianca da Silva Miguel, Igor Paiva Ramos, Aline Cristina Zago, Rosilene Luciana Delariva, Lidiane Franceschini

**Affiliations:** 1 Programa de Pós-graduação em Ciências Biológicas (Zoologia), Instituto de Biociências, Universidade Estadual Paulista (UNESP), Botucatu, SP, Brasil; 2 Laboratório de Ecologia de Peixes – Pirá, Departamento de Biologia e Zootecnia, Faculdade de Engenharia, Universidade Estadual Paulista (UNESP), Ilha Solteira, SP, Brasil; 3 Laboratório de Ictioparasitologia Neotropical – LABIN, Departamento de Ciências Biológicas, Faculdade de Ciências, Universidade Estadual Paulista (UNESP), Bauru, SP, Brasil; 4 Laboratório de Ictiologia, Ecologia e Biomonitoramento, Universidade Estadual do Oeste do Paraná (UNIOESTE), Cascavel, PR, Brasil; 5 Laboratório de Ictiologia, Instituto de Biociências Letras e Ciências Exatas, Universidade Estadual Paulista (UNESP), São José do Rio Preto, SP, Brasil

**Keywords:** Yellow-tailed tetra, ichtioparasitology, fish of stream, Monogenea, Nematoda, Lambari-do-rabo-amarelo, ictioparasitologia, peixe de riacho, Monogenea, Nematoda

## Abstract

*Astyanax lacustris* is a small characid fish widely distributed in Brazil, with fast-growing and omnivorous feeding habits. Although the species presents economic and ecological importance, little is known about its parasitological fauna in stream environments. This study aimed to characterize the parasitic fauna of *A. lacustris* in two streams in the state of Paraná, Brazil. Fifty-two specimens of *A. lacustris* were collected, 22 from the Carolina stream (Lower Iguaçu River) and 30 from the Carreira stream (Upper Paraná River), in July and September 2018. In both streams, there was a low richness of parasites, and the structure of the parasitic community was predominantly composed of monogeneans. These findings may be associated with the gregarious behavior of the host species. Moreover, the low occurrence of endohelminths, may be associated with the fact that in streams, the energy flow is low, and depends directly on the input of allochthonous matter, which favors the formation of shelters for the establishment of macroinvertebrates, which represent important sources of food for the ichthyofauna, and may act as intermediate and paratenic hosts of parasites. These environments require further studies to support conservation measures aimed at maintaining the balance of ecological relationships in these preserved ecosystems.

## Resumo

*Astyanax lacustris* é um caracídeo de pequeno porte, amplamente distribuído no Brasil, com hábito alimentar onívoro e rápido crescimento. Embora a espécie tenha importância econômica, pouco se sabe sobre sua fauna parasitológica em ambientes de riacho. Este estudo objetivou caracterizar a fauna parasitária de *A. lacustris* em dois riachos do estado do Paraná. Foram coletados 52 exemplares de *A. lacustris*, sendo 22 no riacho Carolina (baixo rio Iguaçu) e 30 no riacho Carreira (alto rio Paraná), nos meses de julho e setembro de 2018. Em ambos os riachos, houve baixa riqueza de parasitos e a estrutura da comunidade parasitária foi caracterizada pela predominância de monogenéticos. Esses achados podem estar associados ao comportamento gregário da espécie hospedeira. Ademais, a baixa ocorrência de endohelmintos pode estar associada ao fato de que, nos riachos, o fluxo de energia é baixo e depende diretamente do aporte de matéria alóctone, que favorece a formação de abrigos para o estabelecimento de macroinvertebrados, os quais representam uma das principais fontes de alimento da ictiofauna, e podem atuar como hospedeiros intermediários e paratênicos de parasitos. Esses ambientes necessitam de mais estudos para subsidiar medidas de conservação que visem manter o equilíbrio das relações ecológicas nesses ecossistemas preservados.

## Introduction

Parasites are ubiquitous components of biological systems that have evolved into multiple independent lineages throughout the history of life, resulting in a greater diversity of taxa than their free-living counterparts (Timi & Poulin, 2020). These organisms play significant roles in ecosystems by regulating the abundance or density of host populations, stabilizing food webs, and contributing to the structuring of animal communities (Timi & Poulin, 2020). Thus, parasites directly influence biological diversification processes, host community composition, and ecological interactions such as predation and competition (Gómez & Nichols, 2013).

The structure of parasite communities may indicate the biological aspects of their hosts related to diet, social behavior, distribution, and phylogeny (Timi & Poulin, 2020). Therefore, these organisms can be used to complement studies on the composition of biological communities as an indirect indicator of the diversity of free-living organisms that make up their respective biological cycles (Hechinger et al., 2007).

Despite the relevance of parasites in the balance of ecological relationships and ecosystems and their potential to provide essential ecological information about the environment and their hosts, these organisms are still neglected in many environmental studies (Timi & Poulin, 2020). This fact places them in a precarious position, with only a few studies aimed at measures for the conservation of parasite diversity (Gómez & Nichols, 2013). Although there are several studies on wild fish parasites and new parasite species being described in Brazil (Zago et al., 2018, 2021; Jorge et al., 2022), fish parasites still constitute one of the least studied groups in most biodiversity studies, especially when considering the diverse ichthyofauna (Acosta et al., 2020) and the wide Brazilian water network.

The ichthyofauna of neotropical streams is the richest and most diverse on the planet, consisting primarily of small species (< 15 cm) and fish from the family Characidae (Characiformes) (Castro, 2021). Among characids, *Astyanax* Baird & Girard, 1854 is one of the most specious genera with high morphological and ecological similarity (Terán et al., 2020). *Astyanax lacustris* (Lütken, 1875) (=*Astyanax altiparanae*), popularly known as “tambiú” or “lambari-do-rabo-amarelo” is a small characid widely distributed in South America (Froese & Pauly, 2022), inhabiting different freshwater environments (Negrelli et al., 2018) in the Paraná River basin (Langeani et al., 2007) and the lower Iguaçu basin (Daga et al., 2016). This fish species is considered a generalist omnivore (Negrelli et al., 2018) and is known for its successful environmental colonization, due to its opportunistic habits and reproductive strategies, including being able to grow quickly, mature early, and spawn more than once a year (Sabbag et al., 2011). In addition, *A. lacustris* is economically valuable, being used for human consumption and as live bait for the sport fishing of carnivorous fish (Sabbag et al., 2011).

Regarding parasitological studies, *A. lacustris* has an established parasitic fauna in several freshwater systems, such as rivers (e.g., Sapucaí-Mirim River (Zago et al., 2018; 2021), Batalha River (Negrelli et al., 2018; Zago et al., 2021) and in the Upper Paraná River floodplain (Capparros & Takemoto, 2017). However, there are limited studies related to the knowledge of the parasitic fauna of its characids in streams (Jorge et al., 2022).

Given that streams are environments that are overlooked from a scientific point of view (Dias et al., 2016), especially those related to fish parasites, this study aimed to characterize the parasitic fauna of *A. lacustris* from two streams in the state of Paraná, Brazil, which are considered highly relevant due to endemism and species richness (Larentis et al., 2022).

## Material and Methods

Fifty-two specimens of *A. lacustris* were collected from two streams in the state of Paraná, Brazil. Twenty-two of them were from the Carolina stream (25°7’1.29” S and 53°10’34.81” W) in the municipality of Catanduvas, Lower Iguaçu River basin. The other 30 were from the Carreira stream (24°58’52.07” S and 53°16’15.76” W) in the municipality of Cascavel, Paraná River basin. Both sampling areas have mostly rural surroundings, with the Carreira stream source originating from an environmental preservation area (Parque Ambiental de Cascavel) (Larentis et al., 2022).

Collections were conducted in July and September 2018 using electric fishing. After capture, the collected fish were euthanized in ice water, weighed (g), measured (cm), placed in plastic bags, and kept frozen until laboratory analysis. The fish standard length and mean total mass values are presented as mean ± error deviation, followed by amplitude (minimum and maximum values).

The parasites were collected after an individual examination of the organs (skin, gills, nostrils, heart, liver, stomach, swim bladder, intestines, and eyes) with the aid of a stereoscopic microscope, and subsequently fixed and processed according to Eiras et al. (2006). For specimens of *A. lacustris* from the Carreira stream, some of the hosts were placed in plastic bags and frozen for subsequent parasitological analysis (n = 6 specimens).

The remaining specimens were fixed in 4% formaldehyde. Therefore, in relation to the ectoparasites present in the mucus of the hosts from this sampling area, only the presence or absence of the taxa was assessed. Morphological analyses of the parasites were performed using a computerized image analysis system with Differential Interference Contrast - LAS V3 (Leica Application Suite). Identifications were made to the lowest possible taxonomic level based on identification keys and relevant literature (Thatcher, 2006; Zago et al., 2018, 2021).

The following ecological descriptors of the parasitism were calculated: prevalence (P), mean abundance (MA), and mean intensity of infection/infestation (MII), according to Bush et al. (1997), considering all parasites recorded for each sampling area separately. The prevalence values are presented as percentages, and MA and MII are presented as mean ± standard error, followed by amplitude (minimum and maximum values). Voucher specimens of the hosts of the Carreira stream were deposited in the Ichthyological Collection of Três Lagoas of the Federal University of Mato Grosso do Sul, Campus of Três Lagoas, State of Mato Grosso do Sul, Brazil (CITL 1114). While the voucher specimens of the hosts of Carolina stream were deposited in the Coleção Ictiológica do Nupélia (Núcleo de Pesquisas em Limnologia, Ictiologia e Aquicultura, State University of Maringá) Campus of Maringá, State of Paraná, Brazil (NUP 25173). Voucher specimens of the parasites (*Cacatuocotyle papilionis* [CHIBB 727L–728L]; *Characithecium* sp. [CHIBB 729L–730L]; *Diaphorocleidus neotropicalis* [CHIBB 731L; 732L; 733L; 734L]; *Gyrodactylus* sp. [CHIBB 735L; 736L; 738L; 739L]; Nematoda gen. sp. (larvae) [CHIBB 10627–10628]) were deposited in the Helminthological Collection of the Biosciences Institute of São Paulo State University, UNESP, Campus of Botucatu, state of São Paulo, Brazil.

## Results

In the Carolina stream, the 22 analyzed specimens of *A. lacustris* presented a mean standard length of 7.87 ± 0.34 (2.50–9.50 cm) and a mean total mass of 17.25 ± 1.62 (0.28–29.89 g). Twenty-one specimens were parasitized by at least one helminth taxon (P = 95.45%), totaling 114 parasite specimens collected and distributed in four monogenean taxa (three belonging to Dactylogyridae and one to Gyrodactylidae) (Table 1). There are no records of endohelminths in fish from this stream.

**Table 1:** Host collection site, ecological descriptors of the parasitism (P: prevalence;MII: mean intensity of infection/infestation; MA: mean abundance), site of infection/infestation (SI) of each parasite taxa recorded in *Astyanax lacustris* (Characiformes, Characidae) from Carolina stream (CA), Lower Iguaçu River region and Carreira stream (CR), Upper Paraná River basin, state of Paraná, Brazil.

**Parasite**	**CA**	**CR**	**CA**	**CR**	**CA**	**CR**	**SI**
	**P (%)**	**P (%)**	**MII**	**MII**	**MA**	**MA**	
Monogenea							
*Cacatuocotyle papilionis*	54.54	#	1.66 ± 0.33 (1–4)	^#^	0.90 ± 0.25 (0–4)	#	Body surface
*Characithecium* sp.	4.54	23.33	2.00	1.42 ± 0.20 (1–2)	0.09 ± 0.09 (0–2)	0.33 ± 0.12 (0–2)	Gills
*Diaphorocleidus neotropicalis*	59.09	60.00	3.15 ± 1.13 (1–15)	4.27 ± 1.18 (1–19)	1.86 ± 0.73 (0–15)	2.56 ± 0.80 (0–19)	Gills
*Gyrodactylus* sp.	59.09	#	3.92 ± 0.71 (1–9)	#	2.31 ± 0.58 (0–9)	#	Body surface
Nematoda							
Nematoda gen. sp. (larvae)	-	6.66	-	1.00	-	0.06 ± 0.04 (0–2)	Stomach
Overall	95.45	70.00	5.42 ± 0.89 (1–16)	4.47 ± 1.10 (1–20)	5.18 ± 0.88 (0–16)	3.13± 0.85 (0–20)	

# Parasites that were registered only the occurrence.

The parasite component community in the Carolina stream was mainly composed of *Gyrodactylus* sp., which were the most prevalent (P = 59.09%) and abundant (2.31 ± 0.58 [0–9]) parasites, followed by the dactylogyrids *Diaphorocleidus neotropicalis* Zago, Franceschini, Abdallah, Müller, Azevedo & Silva, 2021 (P = 59.09%; MA = 1.86 ± 0.73 [0–15]) (Table 1).

For the Carreira stream, the 30 specimens of *A. lacustris* had a mean standard length of 7.82 ± 0.13 (6.00–9.00 cm) and a mean total mass of 14.36 ± 1.42 (8.89–27.06 g). Among the 30 fish specimens analyzed, 21 were parasitized by at least one helminth taxon (P = 70.00%), totaling 94 parasite specimens belonging to five taxa: one nematode species in the larval stage and four monogeneans (three belonging to Dactylogyridae and one to Gyrodactylidae) (Table 1). *Diaphorocleidus neotropicalis* was the most prevalent (60.00%) and abundant (2.56 ± 0.80 [0–19]) taxon (Table 1) in the analyzed component community.

## Discussion

This is the first study to report the parasitic fauna of *A. lacustris* in streams for the location of the lower Iguaçu River basin and the upper Paraná River basin. Furthermore, it is the first parasitological record of *A. lacustris* for the lower Iguaçu River basin. The predominance of monogeneans may be associated with two characteristics related to the life cycle and dispersal mode of these organisms. Monogeneans have a monoxenous life cycle, therefore, their transmission occurs mainly through direct contact with their hosts (Thatcher, 2006). Dactylogyrids have larval forms (oncomiracids) that are ciliated and free-swimming, allowing them to swim until they find a new host in the shoal (Eiras et al., 2010). Therefore, the transmission of monogeneans can be facilitated by the gregarious behavior of the hosts (Negrelli et al., 2018), as reported for *A. lacustris* (Camargo et al., 2016). In this case, although the formation of shoals represents a host defense and anti-predation tactic, it can also increase the probability of parasite transfer to another host (Negrelli et al., 2018), especially in those parasites in which transmission occurs through direct contact between hosts.

The monogenean taxa recovered from *A. lacustris* in the present study have previously been reported in characids from other aquatic environments in the Upper Paraná River floodplain. Capparros & Takemoto (2017) reported two species belonging to the Gyrodactylidae (*Gyrodactylus neotropicalis* Kritsky & Fritts, 1970 and *Anacanthocotyle anacanthocotyle* Kritsky & Fritts, 1970) parasitizing *A. lacustris* and *Moenkhausia forestii* Benine, Mariguela & Oliveira, 2009. Recently, Zago et al. (2018, 2021) described new species of dactylogyrids reported in the present study: *Cacatuocotyle papilionis* Zago, Franceschini, Müller & Silva, 2018 (in the Sapucaí-Mirim River) and *D. neotropicalis* (in the Sapucaí-Mirim River and Batalha River). These monogeneans were described as parasitizing characids belonging to the genera *Astyanax* and *Psalidodon* Eigenmann, 1911 from two different localities in the Paraná River basin, State of São Paulo.

The presence of nematode larvae in the stomach suggests two hypotheses to be investigated: 1) *A. lacustris* may accidentally ingest the larvae together with its prey, and/or 2) *A. lacustris* can act as an intermediate or paratenic host in the life cycle of these larval forms. This corroborates previous studies reporting that *A. lacustris* occupies an intermediate position in the food chain of aquatic ecosystems and serves as food for several carnivorous predators (Lizama et al., 2008).

The parasitic community of *A. lacustris* in the two streams evaluated (river basins: Iguaçu and Paraná) showed low helminth richness compared to other water environments such as rivers, lakes, and floodplains (Lizama et al., 2008; Camargo et al., 2016; Negrelli et al., 2018). Camargo reported about 15 parasitic taxa in the Peixe River, state of São Paulo, while Lizama et al. (2008) reported 23 species of parasites in the Upper Paraná River floodplain. This may be related to the inherent characteristics of streams, as they are small lotic watercourses that, despite being more dynamic, present less complexity than other environments such as floodplains, in addition to having a lower system energy flow and limited autochthonous primary productivity (Castro, 2021). In the present study, the low parasite richness in the parasite community of *A. lacustris* and the low occurrence of endohelminths, may be associated with the fact that in streams, the energy flow is low, and depends directly on the input of allochthonous matter (e.g. branches, leaves, seeds, fruits, and sediment) which favors the formation of shelters for the establishment of macroinvertebrates (e.g. crustaceans, molluscs, and insect larva) which represent one of the main sources of food for the ichthyofauna (Bennemann et al., 2005; Silva et al., 2014), and which may act as intermediate and paratenic hosts of parasites (Thatcher 2006).

The present study contributes to the literature on fish parasites from streams, and the knowledge and understanding of the parasite-host relationship in *A. lacustris*. These findings demonstrate that, despite the low parasite richness, there was a predominance of monogeneans in the parasitic fauna of *A. lacustris*, which may be associated with the behavior of the host species and the lower environmental flow of energy of the streams compared to other aquatic environments. Moreover, few studies have explored these environments from a parasitological perspective, and new studies are needed to support conservation measures aimed at maintaining the balance of ecological relationships in these threatened ecosystems.
